# Supplementary material for: A survey on modeling dynamic business processes
Source: PeerJ Comput Sci. 2021 Jul 2;7:e609. doi: 10.7717/peerj-cs.609 (PMC8279139; doi:10.7717/peerj-cs.609)
Supplement: Supplemental Information 1 [file peerj-cs-07-609-s001.docx]

| **Excluded references** | **Reason** |
| --- | --- |
| Arnold, O., & Laue, R. (2015). Overview and further development of methods for the controllability of variability in business process models [überblick und Weiterentwicklung von Methoden zur Beherrschbarkeit von Va riabilität in Geschäftsprozessmodellen]. In S. I. M. K. Cunningham D.W. Hofstedt P. (Ed.), Lecture Notes in Informatics (LNI), Proceedings - Series of the Gesellschaft fur Informatik (GI) (Vol. 246, pp. 993–1005). Gesellschaft fur Informatik (GI). Retrieved from https://www.scopus.com/inward/record.uri?eid=2-s2.0-85018333963&partnerID=40&md5=1e95d5d9886bb11561bbb0410490e906 | excluded, no full text |
| Bentellis, A., & Boufaida, Z. (2009). Objective Based Flexible Business Process Management Using the Map Model. Information Technology Journal, 8(4), 495-503. | excluded, not new |
| Berthier, D. (2007). An ontology for modelling flexible business processes. In Advances and innovations in systems, computing sciences and software engineering (pp. 83-88). Springer, Dordrecht. | excluded, partial description, only ontology |
| Beyer, J., Kuhn, P., Hewelt, M., Mandal, S., & **Weske**, M. (2016, September). Unicorn meets Chimera: Integrating External Events into Case Management. In BPM (Demos) (pp. 67-72). | excluded, small part of all approach |
| Weidlich, M., Smirnov, S., Wiggert, C., & **Weske**, M. (2011). Flexab-Flexible Business Process Model Abstraction. In CAiSE Forum (Vol. 734, pp. 17-24). | excluded, no approach, only prototype implementation |
| Braunwarth, K. S., & Ullrich, C. (2010, June). Valuating Business Process Flexibility achieved through an Alternative Execution Path. In ECIS (p. 24). | alternative paths only not analysed further |
| Burkhart, T., & Loos, P. (2010). Flexible business processes-evaluation of current approaches. Multikonferenz Wirtschaftsinformatik 2010, 243. | excluded, 2 pages of text |
| Cognini, R., Polini, A., Polzonetti, A., & Re, B. (2015, July). BPFM: A Notation and an Approach to Homogenize Variable Business Processes for Public Services. In Advanced Applied Informatics (IIAI-AAI), 2015 IIAI 4th International Congress on (pp. 34-39). IEEE. | excluded, dynamicity based on constraints |
| Yiru, D., & Jian, W. (2006). Variation knowledge-based approach to handling business process changes. | excluded, no full text |
| Daoudi, F., & Nurcan, S. (2007). A benchmarking framework for methods to design flexible business processes. Software Process: Improvement and Practice, 12(1), 51-63. | excluded, not appropriate, not full description |
| Demirkan, H., Kauffman, R. J., Vayghan, J. A., Fill, H. G., Karagiannis, D., & Maglio, P. P. (2008). Service-oriented technology and management: Perspectives on research and practice for the coming decade. *Electronic commerce research and applications*, *7*(4), 356-376. | excluded, not appropriate |
| Desai, N., Chopra, A. K., & Singh, M. P. (2006, September). Business process adaptations via protocols. In *Services Computing, 2006. SCC'06. IEEE International Conference on*(pp. 103-110). IEEE. | excluded, not appropriate |
| Ding, F., & Jie, L. (2008, July). An empirical study of flexible business process based on modularity system theory. In *Computing in the Global Information Technology, 2008. ICCGI'08. The Third International Multi-Conference on* (pp. 37-44). IEEE. | excluded, modularity only discussed as flexibility |
| Dörner, C., Yetim, F., Pipek, V., & Wulf, V. (2011). Supporting business process experts in tailoring business processes. *Interacting with Computers*, *23*(3), 226-238. | excluded, not appropriate |
| Duc, B. L., Châtel, P., Rivierre, N., Malenfant, J., Collet, P., & Truck, I. (2009, November). Non-functional data collection for adaptive business processes and decision making. In *Proceedings of the 4th International Workshop on Middleware for Service Oriented Computing* (pp. 7-12). ACM. | excluded, not appropriate |
| Elliman, T., & Eatock, J. (2005). Online support for arbitration: designing software for a flexible business process. *International journal of information technology and management*, *4*(4), 443-460. | excluded, not appropriate |
| Engelhardt-Nowitzki, C., Kryvinska, N., & Strauss, C. (2011, September). Strategic demands on information services in uncertain businesses: a layer-based framework from a value network perspective. In *Emerging Intelligent Data and Web Technologies (EIDWT), 2011 International Conference on* (pp. 131-136). IEEE. | excluded, requirements, not appropriate |
| Esfahani, F. S., Murad, M. A. A., Sulaiman, M. N., & Udzir, N. I. (2009, February). SLA-Driven Business Process Distribution. In *Information, Process, and Knowledge Management, 2009. eKNOW'09. International Conference on*(pp. 14-21). IEEE. | excluded, process distribution |
| Esfahani, F. S., Murad, M. A. A., Sulaiman, M. N., & Udzir, N. I. (2009, March). Using process mining to business process distribution. In *Proceedings of the 2009 ACM symposium on Applied Computing* (pp. 2140-2145). ACM. | excluded, process distribution |
| Estrada-Torres, B. (2017, September). Improve Performance Management in Flexible Business Processes. In *Proceedings of the 21st International Systems and Software Product Line Conference-Volume B* (pp. 145-149). ACM. | excluded, not full paper |
| Fabra, J., De Castro, V., Álvarez, P., & Marcos, E. (2012). Automatic execution of business process models: Exploiting the benefits of model-driven engineering approaches. *Journal of Systems and Software*, *85*(3), 607-625. | excluded, not appropriate |
| Fabra, J., Peña, J., Ruiz-Cortés, A., & Ezpeleta, J. (2008, June). Enabling the evolution of service-oriented solutions using an UML2 profile and a reference Petri nets execution platform. In *Internet and Web Applications and Services, 2008. ICIW'08. Third International Conference on* (pp. 198-204). IEEE. | excluded, not appropriate |
| Fadahunsi, O., & Sathiyanarayanan, M. (2016, December). Visualizing and analyzing dynamic business process using petri nets. In *Contemporary Computing and Informatics (IC3I), 2016 2nd International Conference on* (pp. 79-84). IEEE. | excluded, visualization Petri nets, not appropriate |
| Fajar, A. N., Budiardjo, E. K., & Hasibuan, Z. A. (2012, April). System architecture in the dynamic environment based on commonality and variability business processes. In *Computing Technology and Information Management (ICCM), 2012 8th International Conference on* (Vol. 1, pp. 465-469). IEEE. | excluded, not appropriate, no full paper |
| Fu, P., Peng, Q., & Hu, X. (2015, May). A web service composition system based on semantic parsing. In *Computer Supported Cooperative Work in Design (CSCWD), 2015 IEEE 19th International Conference on* (pp. 561-569). IEEE. | excluded, SOA, not appropriate |
| Ghilic-Micu, B., Stoica, M., Mircea, M., & Sinioros, P. (2010). Cost-performance evaluation environment for the adoption of a case management solution. *Economic computation and economic cybernetics studies and research*, *44*(1), 63-79. | excluded, cost evaluation, not appropriate |
| Ghoneim, A., Elmougy, S., & Al-Husain, L. (2009, August). Integrative framework for Service Oriented Architecture composition of business processes. In *Applications of Digital Information and Web Technologies, 2009. ICADIWT'09. Second International Conference on the* (pp. 139-144). IEEE. | excluded, SOA, not appropriate |
| Goedertier, S., & Vanthienen, J. (2006, January). Compliant and flexible business processes with business rules. In *7th Workshop on Business Process Modeling, Development and Support (BPMDS'06) at CAiSE'06* (pp. 94-104). Presses Universitaires de Namur. | excluded, BR separation from BP, not appropriate |
| Golani, M., & Gal, A. (2005, September). Flexible business process management using forward stepping and alternative paths. In *International Conference on Business Process Management* (pp. 48-63). Springer, Berlin, Heidelberg. | excluded, not appropriate |
| **Gong**, Y., Janssen, M., Overbeek, S., & Zuurmond, A. (2009). Enabling flexible processes by ECA orchestration architecture. In *Proceedings of the 3rd International Conference on Theory and Practice of Electronic Governance - ICEGOV ’09*(p. 19). New York, New York, USA: ACM Press. https://doi.org/10.1145/1693042.1693048 | excluded, similar to the included article |
| Grambow, G., Mundbrod, N., Steller, V., & Reichert, M. (2013). Challenges of applying adaptive processes to enable variability in sustainability data collection. | excluded, some tipe of summary, without approach |
| Grefen, P., Ludwig, H., Dan, A., & Angelov, S. (2006). An analysis of web services support for dynamic business process outsourcing. *Information and Software Technology*, *48*(11), 1115-1134. | excluded, outsoursing |
| Hacid, H., Ugljanin, E., Sellami, M., & Maamar, Z. (2017). Adapting selection strategies of executors of business processes based on profit and social qualities. *Computers & Electrical Engineering*, *63*, 320-331. | excluded |
| Hajmoosaei, M., Tran, H. N., Percebois, C., Front, A., & Roncancio, C. (2015, June). Impact Analysis of Process Change at Run-Time. In *Enabling Technologies: Infrastructure for Collaborative Enterprises (WETICE), 2015 IEEE 24th International Conference on* (pp. 156-161). IEEE. | excluded, impact analysis, not dynamicity |
| Hildebrandt, T., & Zanitti, F. (2012, July). A process-oriented event-based programming language. In *Proceedings of the 6th ACM International Conference on Distributed Event-Based Systems* (pp. 377-378). ACM. | excluded, 2 pages of text |
| Jain, P., Yeh, P. Z., Verma, K., Kass, A., & Sheth, A. (2008, October). Enhancing process-adaptation capabilities with web-based corporate radar technologies. In *Proceedings of the first international workshop on Ontology-supported business intelligence* (p. 2). ACM. | excluded, specific topic |
| Jeng, J. J., & Chang, H. (2009, July). Cogito: An Adaptive Business Process Framework. In *Services-I, 2009 World Conference on* (pp. 463-464). IEEE. | excluded, 2 pages of text |
| Liu, B., Shen, J. Q., Tan, F., Wang, D. W., Tang, L., & Yan, S. F. (2013). Study on Measurement Method for Flexible Management Effect of Project Schedule under Construction Supply Chain Mode. In *Advanced Materials Research* (Vol. 734, pp. 3366-3369). Trans Tech Publications. | excluded, no full text |
| Kai, C. H., & Liew, S. C. (2012). Applications of belief propagation in CSMA wireless networks. *IEEE/ACM Transactions on Networking*, *20*(4), 1276-1289. | exclude, not appropriate |
| Kherbouche, O. M., Ahmad, A., Bouneffa, M., & Basson, H. (2013, December). Ontology-based change impact assessment in dynamic business processes. In *Frontiers of Information Technology (FIT), 2013 11th International Conference on* (pp. 235-240). IEEE. | excluded, dependency relationship through ontology and BPMN |
| Khoshnevis, S., & Shams, F. (2017). Automating identification of services and their variability for product lines using NSGA-II. *Frontiers of Computer Science*, *11*(3), 444–464. https://doi.org/10.1007/s11704-016-5121-6 | excluded, multi-object optimization problem |
| Kochanowski, M., Renner, T., & Weisbecker, A. (2012). Goal-oriented dynamic business processes in service environments: Models and methods applied in finance industry. In *Annual SRII Global Conference, SRII*(pp. 875–884). https://doi.org/10.1109/SRII.2012.99 | excluded, general descriptions |
| Kolokolov, V. . V., Baumann, P. P. ., Santini, S. . S., Ruehl, S. T. S. T. ., & Verclas, S. A. W. . S. A. W. (2013). Flexible development of variable software features for mobile business applications. *ACM International Conference Proceeding Series*, 67–73. https://doi.org/10.1145/2499777.2500712 | excluded, not full description of an approach |
| Kopetzky, T., & Geist, V. (2012). Workflow charts and their semantics using abstract state machines. In R.-M. S. Weske M. (Ed.), *Lecture Notes in Informatics (LNI), Proceedings - Series of the Gesellschaft fur Informatik (GI)*(Vol. P-206, pp. 11–24). Gesellschaft fur Informatik (GI). Retrieved from https://www.scopus.com/inward/record.uri?eid=2-s2.0-84954487237&partnerID=40&md5=af4c836975b5b2b15abd122729dd2657 | excluded, not appropriate |
| Král, J., & Žemlička, M. (2005). Software architecture for evolving environment. In *Proceedings - 13th IEEE International Workshop on Software Technology and Engineering Practice, STEP 2005*(Vol. 2005, pp. 49–58). https://doi.org/10.1109/STEP.2005.25 | excluded, not appropriate |
| Krupaviciute, A., & Fayn, J. (2010). Ontology driven approach enhancing business services orchestration. In *Proceedings - 2nd International Conference on Computational Intelligence, Communication Systems and Networks, CICSyN 2010*(pp. 344–348). https://doi.org/10.1109/CICSyN.2010.33 | excluded, not full description of an approach |
| Kumar, K., & Narasipuram, M. M. (2006). Defining Requirements for Business Process Flexibility. *BPMDS*, *6*, 137-148. | excluded, no approach |
| La Rosa, M. (2017). Modeling Business Process Variability: Are we done yet? *Proceedings of the 21st International Systems and Software Product Line Conference - Volume A on - SPLC ’17*, *1*, 3–3. https://doi.org/10.1145/3106195.3106196 | excluded, 1 page |
| Lassoued, Y., Bouzguenda, L., & Mahmoud, T. (2016). Context-Aware Business Process Versions Management. *International Journal of E-Collaboration (IJeC)*, *12*(3), 7–33. https://doi.org/10.4018/IJeC.2016070102 | excluded, no full text |
| Duc, B. L., Châtel, P., Rivierre, N., Malenfant, J., Collet, P., & Truck, I. (2009). Non-functional Data Collection for Adaptive Business Processes and Decision Making. In *Proceedings of the 2009 International Workshop on Middleware for Service Oriented Computing*(pp. 7–12). ACM. https://doi.org/10.1145/1657755.1657757 | excluded, not appropraite |
| Lederer, M., Huber, S., & Zellner, C. (2013). Business process deployment scorecard: Managing process changes transparently. In *Proc. of the IADIS Int. Conf. Information Systems Post-Implementation and Change Management 2013, ISPCM 2013, Proc. of the IADIS Int. Conf. Theory and Practice in Modern Computing 2013, TPMC 2013*. | excluded, no full text |
| Lee, J., Seo, W., Kim, K., & Kim, C. H. (2010). An OWL-based ontological approach to RAD modeling of human interactions for business collaboration. *Expert Systems with Applications*, *37*(6), 4128–4138. https://doi.org/10.1016/j.eswa.2009.11.011 | excluded, not appropriate |
| Ning, L., Jianchu, K., & Weifeng, L. (2005). A hybrid approach for dynamic business process mining based on reconfigurable nets and event types. In *Proceedings - ICEBE 2005: IEEE International Conference on e-Business Engineering*(Vol. 2005, pp. 289–294). https://doi.org/10.1109/ICEBE.2005.5 | excluded, mining |
| Liu, H., Charif, Y., Jung, G., Quiroz, A., Goetz, F., & Sharma, N. (2012). Towards simplifying and automating business process lifecycle management in hybrid clouds. In *Proceedings - 2012 IEEE 19th International Conference on Web Services, ICWS 2012*(pp. 592–599). https://doi.org/10.1109/ICWS.2012.88 | excluded, not appropriate |
| Luo, W.-W., & Chen, X.-Y. (2011). A research on flexible business process management system based on knowledge base and semantic web services. In *Proceedings of 2011 International Conference on Electronic and Mechanical Engineering and Information Technology, EMEIT 2011*(Vol. 8, pp. 4289–4292). Networking and Switching Technology State Key Laboratory, Beijing University of Posts and Telecommunications, Beijing, China. https://doi.org/10.1109/EMEIT.2011.6023989 | excluded, not appropriate |
| Machado, I., Bonifácio, R., & Alves, V. (2011). Managing variability in business processes: an aspect-oriented approach. *EA ’11 Proceedings of the 2011 International Workshop on Early Aspects*, (February 2016), 25–30. https://doi.org/10.1145/1960502.1960508 | excluded, not new variability, different application domain |
| Maximov, D., & Ryvkin, S. (2017). Systems smart effects as the consequence of the systems complexity. In K. L. Latkoski P. Cvetkovski G. (Ed.), *17th IEEE International Conference on Smart Technologies, EUROCON 2017 - Conference Proceedings*(pp. 576–581). Institute of Electrical and Electronics Engineers Inc. https://doi.org/10.1109/EUROCON.2017.8011178 | excluded, not appropriate |
| Mei, H., Sun, X., Liu, X., Jiao, W., & Huang, G. (2006). An agent-based approach to composing web services to support adaptable business processes. *Multiagent and Grid Systems*, *2*(4), 383–399. https://doi.org/10.3233/MGS-2006-2406 | excluded, no full text |
| Mejri, A., Ghannouchi, S. A., & Martinho, R. (2017). A guidance system for business process flexibility (pp. 210–217). SciTePress. Retrieved from https://www.scopus.com/record/display.uri?eid=2-s2.0-85025469196&origin=resultslist&sort=plf-f&src=s&st1=A+guidance+system+for+business+process+flexibility&st2=&sid=ecf739f44995d861039fcdc369332f5e&sot=b&sdt=b&sl=57&s=TITLE%28A+guidance+system+for+busines | excluded, no full text |
| Mejri, A., Ghannouchi, S. A., Martinho, R., & Elhadj, F. (2016). Enhancing business process flexibility in an emergency care process. In *2016 IEEE/ACIS 15th International Conference on Computer and Information Science, ICIS 2016 - Proceedings*. Institute of Electrical and Electronics Engineers Inc. https://doi.org/10.1109/ICIS.2016.7550814 | excluded, flexibility need in healtcare |
| Minguez, J., Zor, S., & Reimann, P. (2011). Event-driven business process management in Engineer-to-Order supply chains. In *Proceedings of the 2011 15th International Conference on Computer Supported Cooperative Work in Design, CSCWD 2011*(pp. 624–631). https://doi.org/10.1109/CSCWD.2011.5960183 | excluded, SOA, not appropriate |
| Mohammadi, M. (2017). Combination of Modeling Techniques for Supporting Business Process Architecture. *International Journal on Advanced Science, Engineering and Information Technology*, *7*(3), 1038–1048. https://doi.org/10.18517/ijaseit.7.3.1813 | excluded, modelling techniques |
| Moitra, D., & Ganesh, J. (2005). Web services and exible business processes: towards the adaptive enterprise. *Information & Management*, *42*, 921–933. https://doi.org/10.1016/j.im.2004.10.003 | excluded, web technologies |
| Moser, T., Mordinyi, R., Sunindyo, W. D., & Biffl, S. (2010). Semantic service matchmaking in the ATM domain considering infrastructure capability constraints. In *Canadian Semantic Web: Technologies and Applications*(pp. 133–157). https://doi.org/10.1007/978-1-4419-7335-1_6 | excluded, not full text, not appropriate |
| Mosser, S., Hermosillo, G., Le Meur, A. F., Seinturier, L., & Duchien, L. (2011). Undoing event-driven adaptation of business processes. In *Proceedings - 2011 IEEE International Conference on Services Computing, SCC 2011*(pp. 234–241). https://doi.org/10.1109/SCC.2011.58 | excluded, not appropriate |
| Muenstermann, B., Joachim, N., & Beimborn, D. (2009). An empirical evaluation of the impact of process standardization on process performance and flexibility. In *15th Americas Conference on Information Systems 2009, AMCIS 2009*(Vol. 10, pp. 6865–6877). University of Bamberg, Germany. Retrieved from https://www.scopus.com/inward/record.uri?eid=2-s2.0-79551541140&partnerID=40&md5=6c340ed3789a697a545ff9b0e995e4e7 | excluded, not appropriate |
| Murata, K., Shimizu, H., & Sakamoto, N. (2011). Provisioning of standardized business systems. *Fujitsu Scientific and Technical Journal*, *47*(3), 293–299. | excluded, not appropriate |
| Nurcan, S. (2008). A survey on the flexibility requirements related to business processes and modeling artifacts. In *Proceedings of the Annual Hawaii International Conference on System Sciences*. https://doi.org/10.1109/HICSS.2008.39 | excluded, not appropriate, requirements |
| Oberhauser, R. (2016). A hypermedia middleware for process enactment and adaptation. *International Journal of Software Engineering and Its Applications*, *10*(6), 53–68. https://doi.org/10.14257/ijseia.2016.10.6.05 | excluded, not appropriate, midelware |
| Orantes Jiménez, S. D., Gutiérrez Tornes, A. F., & López Sánchez, M. (2008). Managing change in business processes: Adaptability of information systems [Manejando el cambio en los procesos de negocio: Adaptabilidad de los sistemas de información]. In *CISCI 2008 - Septima Conferencia Iberoamericana en Sistema, Cibernetica e Informatica 5to SIECI 2008, 3er Simposium Internacional en Comunicacion del Conocimiento y Conferencias, CCC 2008 - Memorias*(Vol. 3, pp. 80–85). International Institute of Informatics and Systemics, IIIS. Retrieved from https://www.scopus.com/inward/record.uri?eid=2-s2.0-84907045528&partnerID=40&md5=c62fc0d491f32264fc2547a8b5b36e43 | excluded, no full text |
| Osis, J. (2007). *Analysis of business process flexibility at different levels of abstraction*. (J. Cardoso, J. Cordeiro, & J. Filipe, Eds.), *Iceis 2007: Proceedings of the Ninth International Conference on Enterprise Information Systems: Software Agents and Internet Computing*(pp. 389–396). Retrieved from <Go to ISI>://WOS:000253306800069 | excluded, no full text |
| Ozawa, H. (2009). Designing to attain and maintain business process flexibility. In *2009 IEEE Asia-Pacific Services Computing Conference, APSCC 2009*(pp. 193–198). https://doi.org/10.1109/APSCC.2009.5394124 | excluded, BP definition, no approach |
| Park, J., & Yeom, K. (2011). A Modeling Approach for Business Processes Based on Variability. In *2011 Ninth International Conference on Software Engineering Research, Management and Applications*(pp. 211–218). IEEE. https://doi.org/10.1109/SERA.2011.19 | excluded, not appropriate |
| Paschke, A., & Teymourian, K. (2009). Rule Based Business Process Execution with BPEL +, (September), 588–601. | excluded, not appropriate |
| Patiniotakis, I., Apostolou, D., Verginadis, Y., Papageorgiou, N., & Mentzas, G. (2017). Assessing flexibility in event-driven process adaptation. *Information Systems*. | excluded, not appropraite |
| Pereira12, T., Alencar, F., & Castro, J. BVCCON-TOOL: A Modeling Tool to Support Dynamic Business Process Configuration Approach. | excluded, tool presented, no approach |
| Petersen, K., Bramsiepe, N., & Pohl, K. (2006). Applying variability modeling concepts to support decision making for service composition. In *2nd International Workshop on Service-Oriented Computing: Consequences for Engineering Requirements, SOCCER’06*. https://doi.org/10.1109/SOCCER.2006.1 | excluded, not appropriate |
| Poggi, A., Tomaiuolo, M., & Turci, P. (2007). An agent-based service oriented architecture. In *WOA 2007 - 8th AI*IA/TABOO Joint Workshop “From Objects to Agents”: Agents and Industry: Technological Applications of Software Agents*(pp. 157–165). Retrieved from https://www.scopus.com/inward/record.uri?eid=2-s2.0-67650144667&partnerID=40&md5=3cee6c5400e8957ad4e162afefbaee12 | excluded, not approprite |
| Poggi, A., & Turci, P. (2009). An agent-based bridge between business process and business rules. | excluded, not appropriate |
| Popp, R., & Kaindl, H. (2014). Automated Adaptation of Business Process Models Through Model Transformations Specifying Business Rules. *Ceur-Ws.Org*, *2*. Retrieved from http://ceur-ws.org/Vol-1164/PaperVision09.pdf | excluded, not appropriate |
| Qu, L., Chen, Y., & Yang, M. (2009). The coordination and integration of agile supply chain based on service-oriented technology. In *3rd International Symposium on Intelligent Information Technology Application, IITA 2009*(Vol. 1, pp. 351–354). https://doi.org/10.1109/IITA.2009.94 | excluded, not appropriate |
| Radgui, M., Saidi, R., & Mouline, S. (2013). Design for Reuse in Business Process. *International Journal of Enterprise Information Systems*, *9*(4), 12–27. https://doi.org/10.4018/ijeis.2013100102 | excluded, not appropriate |
| Razavian, M., & Khosravi, R. (2008). Modeling Variability in Business Process Models Using UML. *Fifth International Conference on Information Technology: New Generations (Itng 2008)*, 82–87. https://doi.org/10.1109/ITNG.2008.132 | excluded, variability modeling by UML |
| Regev, G., Bider, I., & Wegmann, A. (2007). Defining business process flexibility with the help of invariants. In *Software Process Improvement and Practice*(Vol. 12, pp. 65–79). https://doi.org/10.1002/spip.301 | excluded, variabilyti modelling by invariants |
| Regev, G., Soffer, P., & Schmidt, R. (2008). Taxonomy of Flexibility in Business Processes Taxonomy of Flexibility in Business Processes. *Business*, (August 2016), 4–7. | excluded, only taxonomy |
| Regev, G., & Wegmann, A. (2006). Business Process Flexibility: Weick's Organizational Theory to the Rescue. In *BPMDS*. | excluded, 4 pages |
| Rekik, M., Boukadi, K., & Abdallah, H. B. (2014). A Context Based Scheduling Approach for Adaptive Business Process in the Cloud. *2014 IEEE 7th International Conference on Cloud Computing*, 948–951. https://doi.org/10.1109/CLOUD.2014.137 | excluded, short description |
| Rekik, M., Boukadi, K., & Ben-Abdallah, H. (2017). An end to end framework for context aware business process outsourcing to the cloud. *Computers and Electrical Engineering*, *0*, 1–12. https://doi.org/10.1016/j.compeleceng.2017.05.009 | excluded, not appropriate |
| Ren, C., Wang, W., Dong, J., Ding, H., Shao, B., & Wang, Q. (2008). Towards a flexible business process modeling and simulation environment. In *2008 Winter Simulation Conference*(pp. 1694–1701). IEEE. https://doi.org/10.1109/WSC.2008.4736255 | excluded, not appropriate |
| Rietzke, E., Bergmann, R., & Kuhn, N. (2017). Adaptive business process visualization for a data and constraint-based workow approach. In *CEUR Workshop Proceedings*(Vol. 1917, pp. 188–199). CEUR-WS. | excluded, not appropriate |
| Rong, H. J., Bao, R. J., & Zhao, G. S. (2014). Model Reference Adaptive Neural Control for nonlinear systems based on Back-Propagation and Extreme Learning Machine. In *2014 IEEE Ninth International Conference on Intelligent Sensors, Sensor Networks and Information Processing (ISSNIP)*(pp. 1–6). https://doi.org/10.1109/ISSNIP.2014.6827703 | excluded, not appropriate |
| Rong, W., Liu, K., & Liang, L. (2009). Personalized web service ranking via user group combining association rule. In *2009 IEEE International Conference on Web Services, ICWS 2009*(pp. 445–452). https://doi.org/10.1109/ICWS.2009.113 | excluded, personalization |
| Rzevski, G. (2013). Multi-agent technology for designing adaptive business processes. In *2013 IEEE/ACIS 12th International Conference on Computer and Information Science, ICIS 2013 - Proceedings*(pp. 83–87). https://doi.org/10.1109/ICIS.2013.6607821 | excluded, 3 pages |
| Sackmann, S., & Syring, A. (2010). Adapted Loss Database – A New Approach to Assess {IT} Risk in Automated Business Processes. *{AMCIS} 2010 Proceedings*. Retrieved from http://aisel.aisnet.org/amcis2010/374 | excluded, no paper |
| Saeedi, K., Zhao, L., & Falcone Sampaio, P. R. (2010). Extending BPMN for supporting customer-facing service quality requirements. In *ICWS 2010 - 2010 IEEE 8th International Conference on Web Services*(pp. 616–623). https://doi.org/10.1109/ICWS.2010.116 | excluded, not appropriate |
| Said, I. B., Chaâbane, M. A., Andonoff, E., & Bouaziz, R. (2015, April). BPMN4V-An Extension of BPMN for Modelling Adaptive Processes using Versions. In *ICEIS (3)* (pp. 258-267). | excluded, no paper |
| Sarabadani Tafreshi, A., Riener, R., & Klamroth-Marganska, V. (2017). Quantitative analysis of externally-induced patterns and natural oscillations in the human cardiovascular response: Implications for development of a biofeedback system. *Biomedical Signal Processing and Control*, *36*, 76–83. https://doi.org/10.1016/j.bspc.2017.03.021 | excluded, not appropriate |
| Sauer, T., Minor, M., & Bergmann, R. (2011). Inverse Workflows for Supporting Agile Business Process Management. *Proceedings of the 6th Conference on Professional Knowledge Management*, *182*, 194–203. | excluded, not appropriate |
| Shan, Z., & Kumar, A. (2012). Optimal Adapter Creation for Process Composition in Synchronous vs. Asynchronous Communication. *ACM Transactions on Management Information Systems*, *3*(2), 1–33. https://doi.org/10.1145/2229156.2229160 | excluded, not appropriate |
| Shi, W., Wu, J., Zhou, S., Zhang, L., Yin, Y., & Wu, Z. (2008). Facilitating the flexible modeling of human-driven workflow in BPEL. In *Proceedings - International Conference on Advanced Information Networking and Applications, AINA*(pp. 1615–1624). https://doi.org/10.1109/WAINA.2008.26 | excluded, not appropriate |
| Sinnhofer, A. D., Höller, A., Pühringer, P., Potzmader, K., Orthacker, C., Steger, C., & Kreiner, C. (2017). Combined variability management of business processes and software architectures. In *Proceedings of the International Symposium on Business Modeling and Software Design (BMSD)*(pp. 36–45). https://doi.org/10.5220/0006527300000000 | excluded, not found |
| De Smedta, J., De Weerdta, J., Serrala, E., & Vanthienena, J. Discovering Hidden Dependencies in Constraint-Based Declarative Process Models for Improving Understandability. | excluded, not appropriate |
| Soffer, P., & Ghattas, J. (2006). Business Process Flexibility in Virtual Organizations. In *BPMDS*. | excluded, not appropriate |
| Stavenko, Y., Kazantsev, N., & Gromoff, A. (2013). Business Process Model Reasoning: From Workflow to Case Management. *Procedia Technology*, *9*, 806–811. https://doi.org/10.1016/j.protcy.2013.12.089 | excluded, only overview, no approach |
| Stecjuka, J., Makna, J., & Kirikova, M. (2008). Best practices oriented business process operation and design. In *CEUR Workshop Proceedings*(Vol. 335, pp. 121–129). | excluded, not appropriate |
| Sun, X., Liu, X.-Z., Jiao, W.-P., Huang, G., & Mei, H. (2006). Rule-based approach to supporting adaptable web service composition. *Jisuanji Xuebao/Chinese Journal of Computers*, *29*(7), 1084–1094. Retrieved from https://www.scopus.com/inward/record.uri?eid=2-s2.0-33747518287&partnerID=40&md5=c06888a9c2756e31630e8b50a96c2248 | excluded, not English |
| Sun, Y., Farwick, M., & Chiu, D. K. W. (2009). Constraint-based Authorization Management for mobile collaboration services. In *SERVICES 2009 - 5th 2009 World Congress on Services*(pp. 22–29). https://doi.org/10.1109/SERVICES-I.2009.79 | excluded, not appropriate |
| Tan, W., Zhang, Z., Zhang, Q., Tang, A., & Wang, T. (2014). A Reliable Composite Service Computation Framework. In *2014 Enterprise Systems Conference*(pp. 91–96). https://doi.org/10.1109/ES.2014.46 | excluded, not appropriate |
| Tan, W., Li, S., & Yang, F. (2005). Zero-time enterprise modeling with component assembly and process model optimization techniques. In *The Fifth International Conference on Computer and Information Technology (CIT’05)*(pp. 1135–1139). IEEE. https://doi.org/10.1109/CIT.2005.203 | excluded, not appropriate |
| Terenciani, M., Paiva, D., Landre, G., & Cagnin, M. I. (2015). BPMN* - A Notation for Representation of Variability in Business Process Towards Supporting Business Process Line Modeling (pp. 227–230). https://doi.org/10.18293/SEKE2015-055 | excluded, not appropriate |
| Termer, F., Nissen, V., & Wessels, N. (2014). Business process flexibility through service-oriented architectures - Basic relationships and further research needs [Geschäftsprozessflexibilität durch Service-orientierte Architekturen - Grundlegende Zusammenhänge und weiterer Forschungsbedarf]. In U. D. S. E. Plodereder E. Grunske L. (Ed.), *Lecture Notes in Informatics (LNI), Proceedings - Series of the Gesellschaft fur Informatik (GI)*(Vol. P-232, pp. 889–900). Gesellschaft fur Informatik (GI). Retrieved from https://www.scopus.com/inward/record.uri?eid=2-s2.0-84922485885&partnerID=40&md5=7609ef769c72c614446230d924083976 | excluded, not full text |
| Tiam, R. T., Seriai, A.-D., & Michel, R. (2015). An operational model of variable business process. In H. S. T. E. Maciaszek L. Maciaszek L. (Ed.), *ICEIS 2015 - 17th International Conference on Enterprise Information Systems, Proceedings*(Vol. 3, pp. 162–172). SciTePress. Retrieved from https://www.scopus.com/inward/record.uri?eid=2-s2.0-84939555303&partnerID=40&md5=04b5fb1beac46062d590a8a422635a9e | excluded, no full text |
| Triaa, W., Gzara, L., & Verjus, H. (2016). Organizational Agility Key Factors for Dynamic Business Process Management. In *2016 IEEE 18th Conference on Business Informatics (CBI)*(pp. 64–73). IEEE. https://doi.org/10.1109/CBI.2016.16 | excluded, not appropriate |
| Truong, T. M., & Lê, L. S. (2017). On business process redesign and configuration: Leveraging data mining classification & outliers and artifact-centric process modeling. In *Proceedings - 2016 International Conference on Advanced Computing and Applications, ACOMP 2016*(pp. 59–66). Institute of Electrical and Electronics Engineers Inc. https://doi.org/10.1109/ACOMP.2016.018 | excluded, not appropriate |
| Turetken, O. (2013). Towards a maintainability model for business processes: Adapting a software maintainability model (position paper). In *2013 IEEE 1st International Workshop on Communicating Business Process and Software Models: Quality, Understandability, and Maintainability, CPSM 2013*. IEEE Computer Society. https://doi.org/10.1109/CPSM.2013.6703083 | excluded, not appropriate |
| Uchitel, S. (2016). Business process adaptation using discrete event controller synthesis. In *Proceedings of the International Workshop on Formal Methods for Analysis of Business Systems - ForMABS 2016*(pp. 3–3). New York, New York, USA: ACM Press. https://doi.org/10.1145/2975941.2990289 | excluded, 1 page |
| Walzer, K., Heinze, T., & Klein, A. (2009). Event Lifetime Calculation Based on Temporal Relationships. In *International Conference on Knowledge Engineering and Ontology Development, KEOD2009*(pp. 269–274). Funchal, Madeira, Portugal. | excluded, not appropriate |
| Wang, C., & Zhao, L. (2010). Intermediate view synthesis based on adaptive BP algorithm and view interpolation. *Journal of Convergence Information Technology*, *5*(10), 72–81. https://doi.org/10.4156/jcit.vol5.issue10.10 | excluded, not appropriate |
| Wang, S., Shen, W., & Hao, Q. (2006). An agent-based Web service workflow model for inter-enterprise collaboration. *Expert Systems with Applications*, *31*(4), 787-799. | excluded, not appropriate |
| Wang, X., Ma, D., Shen, J., Cao, H., Han, Y., & Han, J. (2010). On dynamic BP neural network applied in process-tracking modeling. In *CAR 2010 - 2010 2nd International Asia Conference on Informatics in Control, Automation and Robotics*(Vol. 1, pp. 52–55). https://doi.org/10.1109/CAR.2010.5456779 | excluded, 4 pages |
| Wang, Z., Liu, Z., & Yang, Y. (2010). Design and implementation of flexible E-Government platform based on XML Data-Bus and lightweight MVC execution framework ASSH. In *ICCASM 2010 - 2010 International Conference on Computer Application and System Modeling, Proceedings*(Vol. 5). https://doi.org/10.1109/ICCASM.2010.5620125 | excluded, not appropriate |
| Wei, L., Li, Y. L., Zhao, Q. Y., & Shu, H. P. (2010). Dynamic changing model of workflow process based on adaptive component. *Computer Integrated Manufacturing Systems*, *16*(12), 2603-2610. | excluded, not English |
| Weidmann, M., Koetter, F., Renner, T., Schumm, D., Leymann, F., & Schleicher, D. (2011). Synchronization of Adaptive Process Models Using Levels of Abstraction. In *2011 IEEE 15th International Enterprise Distributed Object Computing Conference Workshops*(pp. 174–183). IEEE. https://doi.org/10.1109/EDOCW.2011.24 | excluded, not appropriate |
| Wieczorek, S., Roth, A., ??tef??nescu, A., & Charfi, A. (2008). Precise steps for choreography modeling for SOA validation and verification. In *Proceedings of the 4th IEEE International Symposium on Service-Oriented System Engineering, SOSE 2008*(pp. 148–153). https://doi.org/10.1109/SOSE.2008.43 | excluded, not appropriate |
| Worley, J. H., Chatha, K. A., Weston, R. H., Aguirre, O., & Grabot, B. (2005). Implementation and optimisation of ERP systems: A better integration of processes, roles, knowledge and user competencies. *Computers in Industry*, *56*(6), 620-638. | excluded, not appropriate |
| Wu, B., Lin, R., Wang, P., & Chen, J. (2016). Dynamic business process generation and verification. In *Proceedings - 2016 IEEE International Conference on Services Computing, SCC 2016*(pp. 836–839). Institute of Electrical and Electronics Engineers Inc. https://doi.org/10.1109/SCC.2016.118 | excluded, not appropriate |
| Wu, H., Yin, B., Zhao, X., & Xiang, G. (2006). A workflow model supporting flexible process based on extensible organization. In *Proceedings of 2006 IEEE Asia-Pacific Conference on Services Computing, APSCC*(pp. 640–645). https://doi.org/10.1109/APSCC.2006.19 | excluded, not appropriate |
| Xia, W., Lan, L., & Junpeng, M. (2014). Research on flexible business process of bank modeling based on EPC. In *Proceedings - 2014 International Conference on Management of e-Commerce and e-Government, ICMeCG 2014*(pp. 54–60). Institute of Electrical and Electronics Engineers Inc. https://doi.org/10.1109/ICMeCG.2014.21 | excluded, not appropriate |
| Xu, B., Xu, L. D., Fei, X., Jiang, L., Cai, H., & Wang, S. (2017). A method of demand-driven and data-centric Web service configuration for flexible business process implementation. *Enterprise Information Systems*, *11*(7), 988–1004. https://doi.org/10.1080/17517575.2016.1150522 | excluded, not appropriate |
| Xu, L., & Qu, S. (2011). A resource information description reference model for ITSGrid. In *Proceedings 2011 International Conference on Transportation, Mechanical, and Electrical Engineering, TMEE 2011*(pp. 303–306). https://doi.org/10.1109/TMEE.2011.6199203 | excluded, 4 pages |
| Xue, H., & Wang, Y. (2009). Workflow model based on stochastic Petri nets and performance evaluation. In *2009 IEEE International Symposium on IT in Medicine & Education*(pp. 245–249). IEEE. https://doi.org/10.1109/ITIME.2009.5236424 | excluded, not appropriate |
| Yang, B., & Wang, H. (2009). Flexible Management Information System Based on Axiomatic Design. *2009 International Conference on Management and Service Science*, 1–4. https://doi.org/10.1109/ICMSS.2009.5305666 | excluded, 4 pages |
| Yang, C. C., Yang, L. B., & Liu, Y. X. (2010). Workflow-based design and implementation of flexible enterprise business. In *ICACTE 2010 - 2010 3rd International Conference on Advanced Computer Theory and Engineering, Proceedings*(Vol. 6). https://doi.org/10.1109/ICACTE.2010.5579401 | excluded, not appropriate |
| Yaxiong, T., Jian, W., Xin, W., Datong, H., & Shuxin, Y. (2007). Knowledge-based flexible business process management. In *IEEE Region 10 Annual International Conference, Proceedings/TENCON*. https://doi.org/10.1109/TENCON.2006.343929 | excluded, not appropriate |
| Yaxiong, T. (2010). BPM exception monitoring based on process knowledge. *2010 IEEE Conference on Cybernetics and Intelligent Systems*, 279–284. https://doi.org/10.1109/ICCIS.2010.5518545 | excluded, not appropriate |
| Yongsiriwit, K., Sellami, M., & Gaaloul, W. (2016). A Semantic Framework Supporting Business Process Variability Using Event Logs. In *2016 IEEE International Conference on Services Computing (SCC)*(pp. 163–170). IEEE. https://doi.org/10.1109/SCC.2016.28 | excluded, not appropriate |
| Zeiner, H., Halb, W., Lernbeiß, H., Jandl, B., & Derler, C. (2010). Making business processes adaptive through semantically enhanced workflow descriptions. In *Proceedings of the 6th International Conference on Semantic Systems - I-SEMANTICS ’10*(p. 1). New York, New York, USA: ACM Press. https://doi.org/10.1145/1839707.1839741 | excluded, 3 pages |
| Zerari, M. (2014). Business process artefact adaptability approach based on artificial immune systems. *International Journal of Information and Communication Technology*, *6*(3–4), 355–368. https://doi.org/10.1504/IJICT.2014.063218 | excluded, not appropriate, not full description |
| Zhai, Y. Z. Y., Su, H. S. H., & Zhan, S. Z. S. (2007). A Data Flow Optimization Based Approach for BPEL Processes Partition. *IEEE International Conference on E-Business Engineering (ICEBE’07)*, 410–413. https://doi.org/10.1109/ICEBE.2007.47 | excluded, not appropriate |
| Zhang, H., Wang, F., Zhang, Y., & Zhou, Z. (2015). Requirement-driven adaptive business processes. In *14th International Symposium on Communications and Information Technologies, ISCIT 2014*(pp. 94–98). Institute of Electrical and Electronics Engineers Inc. https://doi.org/10.1109/ISCIT.2014.7011877 | excluded, not appropriate |
| Zhang, J. L., Yang, Y., Zeng, M., & Yuan-Zhuo, W. (2009). Modeling and performance analysis for flexible workflow supporting task change. In *Proceedings - 2009 International Symposium on Information Engineering and Electronic Commerce, IEEC 2009*(pp. 95–99). https://doi.org/10.1109/IEEC.2009.25 | excluded, not appropriate |
| Zhang, Y., Ni, K., Lu, J., Xu, J., & Xiao, G. (2016). DOGCP: A Domain-Oriented Government Cloud Platform Based on PaaS. In *Proceedings - 2nd IEEE International Conference on Cyber Security and Cloud Computing, CSCloud 2015 - IEEE International Symposium of Smart Cloud, IEEE SSC 2015*(pp. 115–120). Institute of Electrical and Electronics Engineers Inc. https://doi.org/10.1109/CSCloud.2015.25 | excluded, not appropriate |
| Zhile, Z., & Zhenhua, D. (2006). Building business processes or assembling service components: Reuse services with BPEL4WS and SCA. In *Proceedings of ECOWS 2006: Fourth European Conference on Web Services*(pp. 138–147). https://doi.org/10.1109/ECOWS.2006.11 | excluded, not appropriate |
| Zhile, Z., Zhenhua, D., & Jianli, W. (2006). A comprehensive framework for dynamic Web services integration. In *Proceedings of ECOWS 2006: Fourth European Conference on Web Services*(pp. 211–220). https://doi.org/10.1109/ECOWS.2006.1 | excluded, not appropriate |
| Zhu, L., Cai, H., & Jiang, L. (2014). Minson: A business process self-adaptive framework for smart office based on multi-agent. In *Proceedings - 11th IEEE International Conference on E-Business Engineering, ICEBE 2014 - Including 10th Workshop on Service-Oriented Applications, Integration and Collaboration, SOAIC 2014 and 1st Workshop on E-Commerce Engineering, ECE 2014*(pp. 31–37). Institute of Electrical and Electronics Engineers Inc. https://doi.org/10.1109/ICEBE.2014.18 | excluded, not appropriate |
| Weber, B., Reichert, M., & Rinderle-Ma, S. (2008). Change patterns and change support features–enhancing flexibility in process-aware information systems. *Data & knowledge engineering*, *66*(3), 438-466. | change patterns described and comparison, but not all approach |
| Haarmann, S., Podlesny, N. J., Hewelt, M., Meyer, A., & Weske, M. (2015). Production case management: A prototypical process engine to execute flexible business processes. In CEUR Workshop Proceedings (Vol. 1418, pp. 110–114). CEUR-WS. | excluded, 5 pages |
| Rinderle, S., Kreher, U., Lauer, M., Dadam, P., & Reichert, M. (2006, June). On representing instance changes in adaptive process management systems. In Enabling Technologies: Infrastructure for Collaborative Enterprises, 2006. WETICE'06. 15th IEEE International Workshops on (pp. 297-304). IEEE. | excluded, poor description |
| Rong, W., Liu, K., & Liang, L. (2008, July). Association rule based context modeling for Web service discovery. In E-Commerce Technology and the Fifth IEEE Conference on Enterprise Computing, E-Commerce and E-Services, 2008 10th IEEE Conference on (pp. 299-304). IEEE. | excluded, short description |
